# BK virus nephropathy is not always alone

**DOI:** 10.15171/jrip.2016.03

**Published:** 2015-04-24

**Authors:** Haydarali Esmaili, Elmira Mostafidi, Mohammadreza Ardalan, Amir Vahedi, Fariba Mahmoodpoor, Mohammadali Mohajel-Shoja

**Affiliations:** ^1^Department of Pathology, Tabriz University of Medical Sciences, Tabriz, Iran; ^2^Kidney Research Center, Tabriz University of Medical Sciences, Tabriz, Iran; ^3^Pediatric Neurosurgery Unit, University of Alabama, Alabama, USA

**Keywords:** BK virus, Nephropathy, Hemophagocytic syndrome, Thrombotic microangiopathy

## Abstract

**Introduction:** BK virus associated allograft nephropathy (BKVAN) is an important cause of allograft lost that often occurs in the first year of transplantation. The state of over immunosuppression also predispose these patients to various opportunistic viral infection

**Objectives:** This research aimed to study the renal transplanted patients for BK viremia and BKVAN.

**Patients and methods:** This observational study was conducted between January 2013 to December 2014 to study the renal transplanted patients for BK viremia and BKVAN. In our center patients received combination of de-sensitization therapy including antithymocyte globulin (ATG), rituximab (RITU), basiliximab, therapeutic plasma exchange, and methylprednisolone (MTP), in high risks or only MTP therapy in immunologically low risk patients.

**Results:** Of total number of 26 patients (20-52 years, M/F 17/9), seven patients received ATG and seven patient received intensive desensitizing protocols, BKVAN and BK viremia happened in three and two patients in above groups subsequently, only one patient developed BKVAN in low risk group. We also observed; cytomegalovirus (CMV) and parvovirus B19 infection and hemophagocytic syndrome (HPS), thrombotic microangiopathy (TMA) and endocarditis in our patients with BKVAN and BK viremia.

**Conclusion:** Awareness about the possibility of BK virus nephropathy and appropriate immunosuppression minimization are crucial components of management. Consideration of other opportunistic infections and specific syndromes are also very important.

**Implication for health policy/practice/research/medical education:**

Awareness about the possibility of BK virus nephropathy and appropriate immunosuppression minimization are crucial components of management of renal transplanted patients. Consideration of other opportunistic infections and specific syndromes are also very important.

## Introduction

BK virus associated allograft nephropathy (BKVAN) recently has become an important cause of allograft lost, and often occurs in the first year of transplantation (1,2). It is most likely due to over immunosuppression state and alloimmune activation induced immunosuppression (3). These patients often remain asymptomatic and are detected when they experience slow and progressive renal allograft dysfunction. (1). Prevalence of BK viremia within the first year is approximately up to 22% and BKVAN has been reported in up to 10% of kidney transplant biopsies (4,5), while, the prevalence of acute rejection in this period is around 13% (6). In another studies the prevalence of viruria, viremia and BKVAN has been reported as 30%, 13%, and 8%, respectively (1,7) while donor or recipient origin of BKV still was not clear (5). BKVAN is associated with a 50% risk for graft lost (8). Increasing awareness of clinicians and the availability of better diagnostic tools may contribute to higher prevalence of this disease in recent years. The immunosuppressive situation that creates BKVAN is also suitable for many other opportunistic infections and pathophysiologic conditions, such as thrombotic microangiopathy (TMA) and activated macrophage syndrome (HPS). Allograft rejection also complicates this condition. In fact, all above conditions lead to allograft failure, if not considered and treated appropriately.

## Objectives

In this investigation, we aimed to report a group of renal transplant recipient with BK viremia and BKVAN nephropathy in whom we also discovered another viral infections and infection related specific syndromes.

## Patients and Methods

### Study population

In this observational study, we collected the clinical and laboratory data from a group of patients who underwent renal transplantation and surveillance for BK viremia and BKVAN (between January 2013 to December 2014). Our investigation consisted of a group of high risk transplanted candidates who received their second or third transplantation with high panel reactive antibody profile who underwent intensive desensitization protocols. We also had a group of low risk renal transplanted patients in our study. Clinical, biochemical and available renal histologic findings were collected during the study period, and then analyzed. Blood samples of patients were taken during the first, third, sixth months and after one year post-transplantation. Blood samples were obtained after blood centrifugation and kept at -80°C until final study that was conducted for BK virus DNA by quantitative real time PCR (BK RG Kit, Novin Gene Co, Tehran, Iran). Detection of the virus in these specimens is an indicative of an active infection and very imminent renal parenchymal involvement. In all patients urine samples were also collected for BK virus DNA detection.

Low risk patients received three daily dosage of methylprednisolone (MTP) (500-1000 mg/day), mycophenolate mofetil (Cellcept, Roche company 2 g/day), tacrolimus (Prograf, Astellas Company) and oral prednisolone as maintenance therapy. All cadaveric transplants, and first renal transplant receipts with more than 10%-30% panel reactivity received antithymocyte globulin (ATG), during the early days of transplantation. Patients with second transplant with higher that 10% panel reactive antibody received an intense desensitization protocol including; every other days of plasmapheresis and intravenous immunoglobulin (IVIG) started from two weeks before transplantation followed by induction therapy with rituximab (administered at 375 mg/m^2^on the evening of transplantation), and IL-2 receptor antagonist basiliximab at morning day of transplantation. Glucocorticoids and ATG were also administered preoperatively like previous group. Tacrolimus trough levels were kept between 8–10 ng/ml at 1 month, 7–8 ng/ml at 3 months, 5–8 ng/ml at 6 months, and 4–7 ng/ml at 1 year. BKVAN suspicious diagnosis was based on blood real-time PCR (RT-PCR) result >10000/µl. Diagnostic kidney biopsy was conducted for evaluation of graft dysfunction and distinguishing the BKVAN with rejection. Antibody-mediated rejection and cellular rejection were treated by MTP/ATG and plasmapheresis. BKVAN were treated with immunosuppressive dose reduction. Intravenous immunoglobulin and recommended antivirals for BK virus.

### Ethical issues

1) The research followed the tenets of the Declaration of Helsinki; 2) informed consent was obtained, and they were free to leave the study at any time; and 3) the research was approved by the ethical committee of Kidney Research Center, Tabriz University of Medical Sciences. In this study we did not introduce any new therapeutic or diagnostic protocols for any conditions. We just observed, collected and analyzed the clinical and laboratory data that was going on in our transplantation unit in a group of renal transplant recipients who received their standard therapeutic and diagnostic measurements in a defined period of time.

### Statistical analysis

Descriptive statistics were used to define the frequencies, means, and medians of study variables.

## Results

Demographic characteristics of our studied patients have been shown in Table 1. Of 26 patients, six had high panel reactive antibody (PRA>50%, one patient), or were recipients of second (5 patients) and third (one patient) renal transplantation with PRA>10% (5 patients) who, received intensive de-sensitization therapy including ATG, RITU (rituximab), basiliximab, therapeutic plasma exchange, and MTP. Seven patients received ATG induction therapy because of cadaveric renal transplantation (one patient), delayed graft function (DGF, 2 patients) and early rejection (3 patients) (Tables 1 and 2).

**Table 1:** Clinical and laboratory characteristics of the studied patients

**Number of patients**	**All**	**Negative BK in plasma**	**Positive BK in plasma**
Number/age	26/(20-52) years	20	‏6
Male/female	17/9	13/7	4/2
Source			
Living related	1 (3.8%)	1/20 (5%)	0
Living unrelated	22 (84.6%‏)	17/20 (85%)	5/6 (83.3%)
Deceased	3 (11.5‏%)	2 /20 (10%)	1 (16.6%)
Induction therapy			
MTP	12/26 (46.1‏%)	11/20 (55%)	1/6 (16.6%)
ATG/MTP	7/26 (26.9%)	4 /20 (20%)	3/6 (50%)
ATG/RITU/BASILI/TPE/MTP‏	7/26 (26.9%)	5/20 (25%)	2/6 (33.3%)
Maintenance therapy‏			
Cyclosporine	3/26 (11.5%)	2/20 (10%)	1/6 (16.6%)
Tacrolimus	23/26 (88.4%)	18/20 (90%)	5/6 (83.3%)
DGF	3/26 (11.5%)	1/20 (5%)	2/6 (33.3%)
Rejection‏	4/26 (15.4%)	1/20 (5%)	3/6 (50%)
Tac B- levels >6			
1st‏ month	23/26 (88.4%)	17/19 (89.4%)	5/6 (83.3%)
3rd‏ month	20/26 (76.9%)	15/20 (75%)	6/6 (100%)
6th‏ month	22/26 (84.6%)	15/20 (75%)	6/6 (100%)
‏12th month	19/26 (73.1%)	14/0 (70%)	5/6 (83.3%)
Time of measurements	First month	Third month	Sixth month
BK viremia detection	1+	4+	1+

Abbreviations: ATG, antithymocyte globulin; RITU, rituximab; BASILI, basiliximab; TPE, therapeutic plasma exchange; MTP, methylprednisolone; DGF, delayed graft function; Tac B- levels, tacrolimus blood levels.

**Table 2:** Comorbid infection and superimposed condition in a group of patients with BK virus viremia

**No.**	**Age/sex**	**Induction**	**CMV/parvovirus B19**	**TMA**	**HPS**	**Other**	**Outcome**
1	39/M	ATG/RITU/BASILI/PE/MTP	-/+	+	+	-	PI
2	48/M	MTP	-/-	-	-	-	ESRD
3	30M	ATG/MTP	-/+	-	-	-	CI
4	52/M	ATG/MTP	+/-	-	-	EnCRD	Death
5	33F	ATG/MTP	-/+	-	+		PI
6	35/M	ATG/RITU/BASILI/PE/MTP	-/-	-	-		CI

Abbreviations: HPS, hemophagocytic syndrome; TMA, thrombotic microangiopathy; ATG, antithymocyte globulin; RITU, rituximab; BASILI, basiliximab; PE, plasma exchange; MTP, methylprednisolone; EnCRD, infective endocarditis; PI, partial improvement (at least 50% reduction of serum creatinine level); CI, complete improvement (serum creatinin level <1.5 mg/dL); CMV, cytomegalovirus infection.

Maintenance immunosuppressive therapy included tacrolimus, prednisolone and mycophenolate mofetil (MMF) in majority of our studied population (23/26, 88.4%), while only 3 patients received cyclosporine A containing immunosuppressive protocol. None of patients received m-TOR inhibitor. Majority of cases of BK viremia and BKVN happened in high risk patients who received intensive desensitizing immunosuppressive regiment containing ATG (5/6 patients). Viremia and BKVN was detected in 5/14 (36%) of all patients who received ATG receivers and only in one patient (1/12, 8%, *P*>0.05) who did not receive ATG. Only one patient in MTP induction therapy developed BKVAN. We performed renal biopsy only in three of patients and we defined BKVN as positive viremia (>10000/µl) combined with compatible pathologic changes including tubulointerstitial nephritis, tubular epithelial cells enlargement and intra-nuclear viral inclusions. Immunohistochemistry staining for SV40 antigen that cross-reacts with BKV infected tubular cells was only performed in one patient (it was performed in another center). The peak incidence of BK virus detection was in third months after transplantation. All patients with significant viremia also had significant viruria (> 10000000/µl).

Differentiate between BKVAN and rejection is a challenge but we were unable to precisely rule out the presence of rejection in this 6 patients. However, clinical condition, improvement of viremia and viruria and allograft function after BK virus directed treatments all were in favor of BKVAN. Interestingly two of our patients who received very intensive desensitization protocol developed BKVAN. All of our patients who received ATG were under CMV prophylaxis with intravenous ganciclovir followed by oral valganciclovir (Valcyte, Roche Company) for 6 months. However, only 1 patient who became CMV positive did not receive oral prophylaxis properly and this patient died because of infective endocarditis and massive splenic infarct. Interestingly we found parvovirus B19 presented with severe anemia in 2 patients, and another parvovirus B19 positive patient had a combination of hemophagocytic syndrome (HPS), and TMA. We also had one case of graft loss as a direct consequence of BKVAN and it was among low risk patients who did not receive ATG. Immunosuppression reduction in BKVAN, is associated with an increased risk for subsequent rejection. We observed this condition in one patient who had a combination of BKVN, HPS and TMA. We, therefore, started treatment with IVIG, leflunomide, plasma exchange, and immunosuppression reduction. After one month his BK viremia resolved and renal function partially improved.

## Discussion

What we observed in this study was the high coincidence of BKVAN and BK viremia with other viral and bacterial infection and specific conditions that rarely happens in immunosuppressed patients. This situation creates a complex clinical picture. We detected the coexistence of parvovirus B19 with severe anemia, CMV infection, TMA, HPS, and endocarditis. These conditions were diagnosed either antecedent, precedent or coexistent with the diagnosis of BKVAN and BK viremia. Our patients also received the specific treatment for above conditions. We think that periodic measurements of viremia and viruria were extremely effective in early detecting of infection. IVIG has neutralizing properties against BK virus. We think the lower incidence of BK viremia and BKVAN in those who received intensive desensitizing regimen containing IVIG compared to those who only received ATG was because of this anti- BK virus activity of IVIG (5,9).

We also used IVIG for treatment of TMA and HPS or those with parvovirus B19 infection and associated KBVAN and BK viremia. We performed a 30%–50% dose reduction of mycophenolate and tacrolimus. It seems that, our practice of reducing both antimetabolite and calcineurin inhibitor was successful in patients with BK viremia and biopsy proven BKVN. Later group additionally received IVIG, leflunomide and ciprofloxacin. No signiﬁcant decline in renal allograft function was detected in patients with BK viremia over one year of reducing immunosuppressions. We also converted two of patients from tacrolimus to cyclosporine, but the clinical beneﬁt of this conversion has not been formally tested (10,11). To date, there is no effective antiviral therapy for BKVAN and its management mainly relies on reducing the total immunosuppression and administration of leﬂunomide yielded mixed results. Additionally administration of cidofovir treatment has been attempted. However its nephrotoxicity is a big concern (10,12). The incidence of BKVAN could be higher than what we detected in our study as there are some reports of BK VN despite negative BK viremia or viruria while, we are not performing protocol biopsies in our center, and hence we are not aware about this condition exactly (1).

Higher tacrolimus plasma level is associated with a greater risk for BKVAN. It has been shown that tacrolimus levels below 6 ng/ml had significantly higher BKV large T-antigen specific activity and IFN-γ release and consequently lower incidence of BKVAN (13). IFN-γ enzyme-linked immunosorbent spot (ELISPOT) assay also is a useful tool to understand the level of anti-BK defense (14). Higher BKV-DNA copy number is associated with an increased likelihood of having nephritis and has also been correlated with severity of disease (1,15).

Urine cytology for decoy cells detection, checking for viruria, or urine VP-1 mRNA every 3 months up to 2 years is also recommended if the test results became positive quantification of DNA load in the urine (threshold >10000000 copies/ml), or plasma DNA load (threshold >10000 copies/ml) has been recommended. If one or more of these tests became positive, then an allograft biopsy is recommended. Screening for quantitative BKV-DNA in plasma at 1, 3, 6, 12, and 24 months after transplantation is another recommendation and we followed the later one (1,4,16).

Major renal allograft involvement in BKVAN is tubulointerstitial nephritis. However, patchy interstitial involvement is another cause of sampling error and false negative results. Moreover, it has a great histologic mimicry with cellular rejection. Even tubular basement membrane C4d staining has been reported in severe BKVAN. Immunohistochemical staining of anti–HLA-DR, and higher percentage of CD20+ cells infiltrates is in favor of acute rejection (17). Morphologic features, of BKVAN including tubular epithelial cells enlargement and intra-nuclear inclusions. Immunohistochemistry staining for SV40 antigen is also diagnostic (18).

## Conclusion

The key point in management of BKVAN requires increasing awareness about it. Awareness to minimize of immunosuppression is in appropriate time before any irreversible changes in renal parenchyma. BKV replication assessment at regular intervals is mandatory particularly during at least the first year after renal transplantation. Particularly in those patients with intense immunosuppression. Keeping the lower targeted trough levels of tacrolimus also is very important. Considering the other opportunistic viral and specific condition in patients with BK viremia and BKNAN is also another very important consideration (17-22).

## Limitations of the study

The study had some limitations such as small sample size and short duration of follow-up, thus we recommend to conduct of similar studies as multi-centric with longer duration of follow-up.

## Acknowledgments

We would like to thank our nephrologist colleagues; Dr. Sima Abediazar, Dr. Jalal Etemadi, Dr. Javid Safa, Dr. Sadredin Hashemi and Dr. Hamid Tayebi Khosroshahi in renal transplantation unit of Imamreza hospital who helped us to have access to the clinical and laboratory data in our observational study.

## Authors’ contribution

MRA; study design, preparation of manuscript and final revision. HE and EM; study design and pathology interpretation. AV and FM; data gathering and data interpretation. MMS; edit of the manuscript.

## Conflicts of interest

The authors declared no competing interests.

## Ethical considerations

Ethical issues (including plagiarism, data fabrication, double publication) have been completely observed by the authors.

## Funding/Support

None.
